# Deciphering barley’s stress response: metabolomic strategies and phenotypic implications under multiple abiotic stresses

**DOI:** 10.1007/s11306-026-02406-8

**Published:** 2026-03-07

**Authors:** Michał Kempa, Anetta Kuczyńska, Piotr Ogrodowicz, Paweł Krajewski, Łukasz Marczak, Martyna Michałek, Krzysztof Mikołajczak

**Affiliations:** 1 Institute of Plant Genetics, Polish Academy of Sciences, Strzeszyńska 34, 60-479 Poznan, Poland; 2 Institute of Bioorganic Chemistry, Polish Academy of Sciences, Noskowskiego 12/14, 61-704 Poznan, Poland

**Keywords:** Barley (*Hordeum vulgare* L.), Multiple abiotic stress, Genotype-dependent stress response, Metabolic biomarkers of stress tolerance, Osmotic adjustment and ROS scavenging, Untargeted metabolomics

## Abstract

**Introduction:**

Plants are mainly influenced by abiotic stresses acting in combination rather than a single stress acting alone. In the present study leaf metabolomic profiles as well as changes in phenome and yield of four barley (*Hordeum vulgare* L.) genotypes of different origin under single and combined abiotic stresses were investigated.

**Objectives:**

The aim of the study was to understand the response of barley to single and combined abiotic stresses and to identify metabolic pathways associated with yield components under stress conditions.

**Results:**

We found that Syrian genotype can be a donor of early resistance caused by rapid increase of amino acids (e.g. proline) under stress, constituting a valuable genetic source in barley breeding. We demonstrated that impact of combined stresses was generally based on the unique response in terms of the metabolomic alterations. However, there were also metabolites that increased their content regardless of the genotype. Methionine sulfoxide has accumulated under long-term drought, salinity and their combination; on the other hand, accumulation of other metabolites such as leucrose increased in drought, but not under its combination with salinity.

**Conclusion:**

Accumulation of most of analysed metabolites significantly depended on the genotype, type of stress as well as their interaction. We indicate that several of identified metabolites might serve as a stress biomarkers (e.g. aspartic acid). We observed that greater phenotypic changes are most visible under the influence of combined stresses being mostly synergistic/additive when compared to single ones as well as considerable relationship between accumulation of specific metabolites and some phenotypic traits.

**Supplementary Information:**

The online version contains supplementary material available at 10.1007/s11306-026-02406-8.

## Background

Abiotic stresses such as drought, salinity and temperature fluctuations strongly limit plant growth and survival, restricting food production to growing human population. These stresses act predominantly together in natural conditions (Sugumar et al., 2024). Global agriculture faces increasing threats from climate change, which amplifies environmental stresses impacting barley - a crop of significant economic importance and the fourth most produced cereal worldwide (Ogrodowicz et al., 2017; FAOSTAT, 2020). Plants developed various ways to counteract stress conditions, but their mechanisms are not fully elucidated (Temel et al., 2017). One of them is the stress escape strategy involving shortening plant’s life cycle to ensure that stress events do not coincide with generative growth (Ogrodowicz et al., 2023). This mechanism is usually utilized by plants originating from arid regions (Franks et al., 2007). Another strategy is stress tolerance involving osmotic adjustment (OA) and accumulation of osmoprotectants such as carbohydrates, organic acids, amino acids, and others (Prabhavathi et al., 2022). These compounds play role in maintaining continuity of cell membranes, stress signalling, gene expression regulation, reactive nitrogen/oxygen species (RN/OS) scavenging, and regulation of ion channels (Roychoudhury et al., 2011; Shevyakova et al., 2013).

**Fig. 1:**
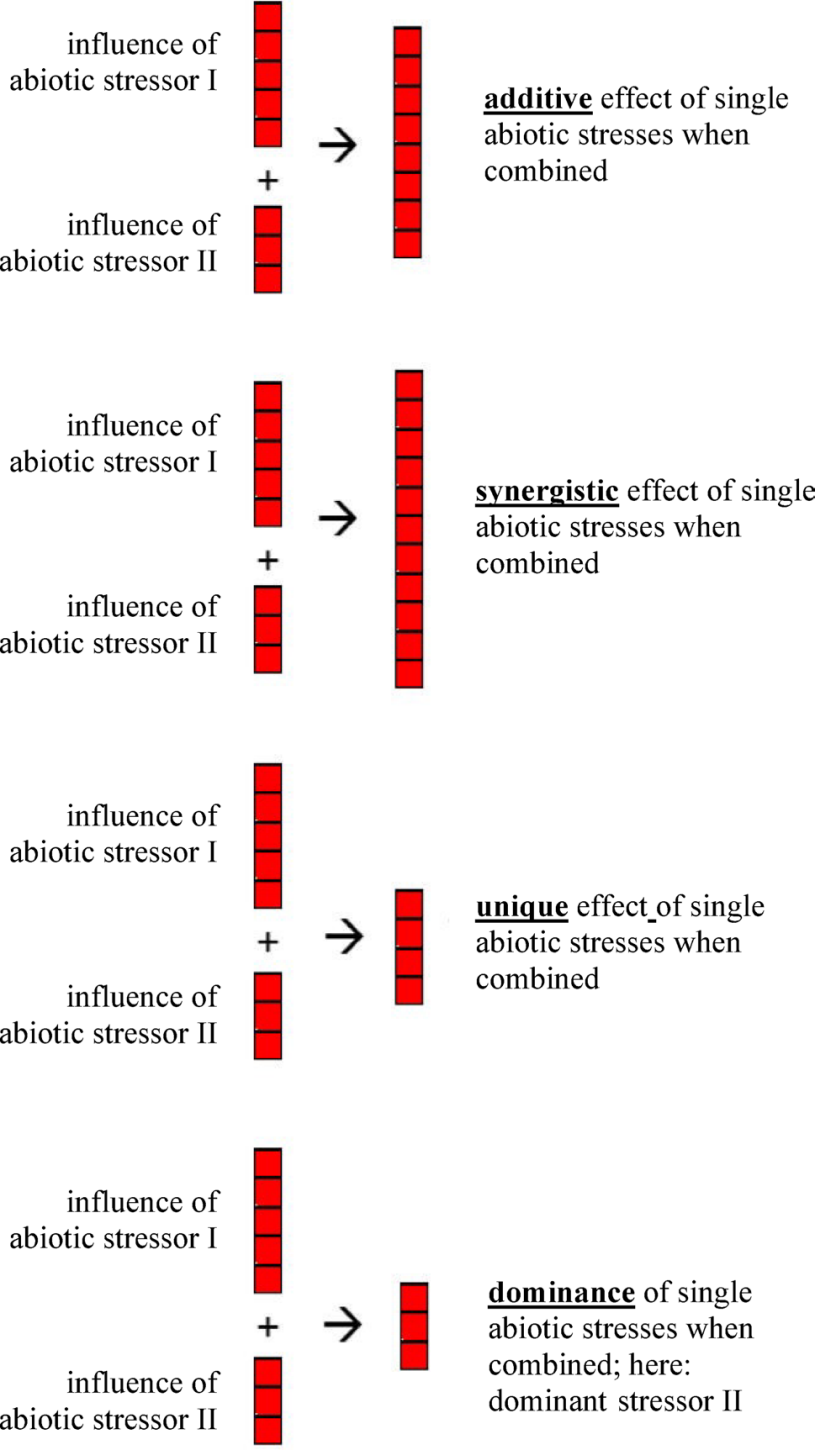
Four types of plant response to combined stresses (Prasch & Sonnewald, 2015)

Plant resilience under adverse conditions fundamentally relies on metabolic adaptation (Liu et al., 2020). Although numerous metabolic pathways in variety of plant species were described under environmental stresses, their better understanding requires further investigation. Primarily, strategies underlying barley response to multiple abiotic stresses remain enigmatic. Prasch and Sonnewald (2015) proposed four types of response by which plants respond to combinatorial environmental stresses, namely (i) additivity, when the effect of a combined stress is equal to the sum of effects of stresses acting separately; (ii) synergistic influence of stresses when the reaction against combined stresses is more than the sum of the effects of individual stresses; (iii) idiosyncratic (unique) response when the response to combined stresses is completely different from single stress responses; (iv) dominance of one of the stresses, when the response to combined stress is very close to the response to one of them (Fig. 1).

It is suggested that the most common strategy involves a unique response, leading to a phenomenon known as cross tolerance (Zandalinas et al., 2020). Generally, combination of stresses has modifying effect on the accumulation of canonical protective compounds in plant leaves. For instance, when drought and high temperature coexist, the accumulation of proline, an amino acid with a well-known protective role under stress conditions, in *A. thaliana* does not reach the same level as when drought acts alone, being lower under combined stress (Rizhsky et al., 2004). Meanwhile, barley and tobacco plants exposed to the same combined stresses had increased proline accumulation in leaves. These examples suggest that proline accumulation under multiple stresses may be species-dependent (Cvikrova et al., 2013; Templer et al., 2017). Regardless of whether the plant is affected by a single or combined abiotic stress, reactive oxygen species (ROS) are produced at varying intensities. While they play an important role in stress signalling, excessive accumulation leads to substantial cellular damage (El Sabagh et al., 2019). Therefore, plants have developed mechanisms of ROS scavenging to limit their toxicity, for instance, accumulation of cytosolic ascorbate peroxidase utilizing ascorbate in ROS scavenging as well as accumulation of other compounds acting in ROS scavenging as low-molecular-weight metabolites, sugars, and others (Keunen et al., 2013; Suzuki et al., 2014; Mansoor et al., 2022).

Generally, the stress-induced metabolic response depends on the type and intensity of the stress. Metabolomic modifications are not only affected by the stressor but are also genetically determined; therefore, the impact of environmental and genetic factors on metabolite profiles were verified in this study. We aimed to determine which of the response types predominates, based on the metabolic reactions observed in barley leaves under combined stress conditions. We hypothesized that genotypes originating from the dry climate may harbour effective mechanisms for adapting barley to abiotic stresses at the early stage of plant development. To gain deeper insights into these processes, we employed untargeted metabolic profiling, identifying key components of biochemical adaptations to drought, salinity, and temperature stress. The study aimed to understand the metabolomic responses of barley to single and combined abiotic stresses and to define metabolic pathways associated with yield components under these conditions. Changes in metabolite accumulation in the leaves of diverse barley genotypes, together with their phenotypic plasticity, were examined to identify early metabolic signatures linked to long-term stress consequences and to pinpoint metabolites that could serve as biomarkers of abiotic stress resistance.

## Methods

### Plant material and stress treatment

The study involved three European barley genotypes: Maresi and two homozygous lines (MPS106, MPS37) derived from Maresi and Pomo cultivars through backcrossing, as well as the Syrian landrace CamB1 line, which served as a reference for the metabolic response to abiotic stress resulting from adaptation to arid climates (Mikołajczak et al., 2017; Kuczyńska et al., 2019; Ogrodowicz et al., 2023). This plant material has been described in detail in our previous studies (Mikołajczak et al., 2016; Kuczyńska et al., 2019; Kempa et al., 2024). Briefly, Maresi is a malting variety with reduced height, MPS106 displays a prostrate juvenile growth habit and carries the recessive semi-dwarf allele *sdw1.d* (3 H), whereas MPS37 is characterized by an erect growth habit and normal height, possessing the dominant *Sdw1.d* allele (Kuczyńska et al., 2013). Additionally, these two lines exhibit polymorphism in the *ns-LTP2.8* gene (located in chromosome 4 H). Syrian CamB1 (Cam/B1/CI08887/CI05761) line is known for its early maturity, and employing a stress escape strategy (Ogrodowicz et al., 2023).

Plants were cultivated in phytotron in three pots per genotype per treatment containing 4.5 kg of soil in which 10 seeds of each genotype were sown and after germination their number was reduced to 5. Field water capacity (FWC) was determined gravimetrically by fully saturating the pots with water and allowing excess water to drain freely overnight. The pot weight after drainage was taken as 100% FWC. During the experiment, soil moisture was maintained at the required percentage of FWC by regular pot weighing and adjusting water supply accordingly, following standard procedures for pot-based drought studies (Puértolas et al., 2017). Soil moisture was monitored daily by weighting and using hand-held FOM/mts device and the appropriate level of FWC and salinity status was maintained (Mikołajczak et al., 2017). The same FOM/mts device was used to evaluate the salinity status of the soil. The experimental conditions and stress application followed our previously established methodology (Kuczyńska et al., 2019; Kempa et al., 2024). Briefly, abiotic stresses began at the tillering stage (21 BBCH) and lasted 14 days. In control conditions (C) 70% FWC was maintained with temperature of 22/18°C during the day/night, air humidity of 50–60% and a photoperiod of 16/8 h, in drought (D) 20% FWC was kept (after 14 days optimal FWC was restored). Salinity (S) was applied by watering plants with an NaCl aqueous solution to obtain final concentration of 250 mM⋅dm^− 3^ in the soil. Temperature stress (T) was established by temperature of 30/10°C day/night. The combinatorial stress conditions (DS, DT, ST and DST) have been achieved by combining single stresses. All of mentioned stress conditions along with control were conducted simultaneously; variants with and without temperature stress were conducted in adjacent phytotrons under identical settings, differing only in temperature. The experiment was arranged in a completely randomized design with three pots per treatment and genotype, and their positions were regularly rotated to minimize positional effects.

Leaves for metabolomic profiling were collected in triplicate at three time points (TP) during stress: TP1–7th, TP2–10th and TP3–14th day. Sampled material was immediately placed in liquid nitrogen and stored at −70 °C.

### Metabolite extraction and profiling

Metabolite extraction and untargeted metabolite profiling were performed according to Chmielewska et al. (2016), in three biological and three technical replicates (100 mg of leaf tissue used per isolation), resulting in nine samples tested by GC-MS of each genotype in given environmental conditions. Instrumental analysis was carried out using gas chromatography coupled with mass spectrometry (GC-MS, TRACE 1310 GC chromatograph with a triple quadrupole MS detector TSQ8000) in the positive ionization mode. After converting raw GC-MS data to an open format (.abf), MSDial (v3.96) was used to treat the data using an untargeted metabolomics method. The following parameters were applied: mass accuracy tolerance 0.5 Da, minimum peak height 700, and deconvolution settings of sigma window value 0.5 with an EI spectra cut-off of 10. For alignment, retention index (RI) tolerance was set to 20. Metabolite annotation was performed by library matching using a minimum match score threshold of 70 (together with RI agreement based on the n-alkane series). Before analysis, plant extracts were chemically derivatized using MSTFA. The built-in GC-MS deconvolution technique in MSDial was used to identify and deconvolve peaks. Next, the retention-time alignment was applied to all samples to produce a feature table containing data from all samples. As an additional constraint during compound annotation, retention indices (Kovats RI) were calculated from a homologous series of n-alkanes (C_10_-C_36_) analysed under the same chromatographic conditions to eliminate shift in retention time (Rt). By comparing experimental EI spectra and retention data to spectrum libraries, i.e., “All records with Kovats RI,” MSP library was annotated, and presumed identifications were made based on spectral similarity and RI agreement. The analytical procedure included pooled quality control (QC) samples as well as blank samples to enable signal normalization and monitor analytical stability. To evaluate background and potential carryover/contaminants, blanks were injected at the start and end of the sequence. Throughout the batch, QC samples (from all sample pools) were injected at regular intervals (every 6 study samples) to monitor instrument performance and time-dependent drift. To avoid systematic biases, reduce the probability of false-positive findings, and reduce potential batch effects, study samples were injected in a randomized order. Features coming from derivatization reagents, column bleed, or laboratory contaminants were eliminated using blank-based filtering. In particular, before further statistical analysis, characteristics with high abundance in blanks compared to biological samples (based on a predetermined blank-to-sample ratio threshold) were eliminated. QC-based normalization was used to correct injection-order effects and signal drift. In order to improve comparability throughout the sequence, feature intensities were normalized using LOWESS (locally weighted scatterplot smoothing) fitted to the repeated QC injections throughout the run. The resulting correction factors were then applied to all samples. The CompMS database was used to identify given compound. Identified artifacts were excluded from further analysis. For further statistical analysis, the processed dataset (aligned peak areas after blank removal and LOWESS correction) was used. The normalized GC-MS data, including retention times and the ions quant mass used for quantification and annotation of detected metabolites are provided in S1 Table.

### Phenotypic analysis

Mature plants were harvested and subjected to biometric analysis of 15 traits (Table 1) in triplicate. Each replication consisted of five plants per pot, with a total of three pots for each genotype and stress condition. Both the main and lateral stems were subjected to evaluation.

**Table 1:** Analysed phenotypic traits (T1–T15)

abbrev.	trait	unit	description
T1	total number of tillers	pcs	number of tillers with fertile and non-fertile spikes-average for 10 plants
T2	number of productive tillers	pcs	number of tillers with fertile spikes-average for 10 plants
T3	length of the main stem	cm	length of the stem from ground level to the end of spike (without awns)-average for 10 main stems
T4	length of the lateral stem	cm	length of the stem from ground level to the end of spike (without awns)-average for 10 lateral stems
T5	length of the main spike	cm	length of main spike-average for 10 spikes from the main stem
T6	number of spikelets per main spike	pcs	number of spikelets in spike of the main stem-average for 10 spikes
T7	number of grains per main spike	pcs	number of grains collected from spike of the main stem-average for 10 spikes
T8	weight of grains per main spike	g	weight of grain collected from one spike of the main stem-average for 10 main spikes
T9	length of the lateral spike	cm	Length of spike from lateral stem-average for 10 lateral spikes (without awns)
T10	number of spikelets per lateral spike	pcs	number of spikelets per spike of lateral stem-average for 10 lateral spikes
T11	number of grains per lateral spike	pcs	number of grains collected from spike of lateral stem-average for 10 lateral spikes
T12	weight of grains per lateral spike	g	weight of grain collected from one spike of the lateral stem-average for 10 lateral spikes
T13	grain yield	g	average weight of grains collected from one plant, calculated as average of measurements of grain weight for 10 plants
T14	thousand grain weight	g	average weight of 1000 grains, calculated as average of 1000 × average weight of one grain for 20 spikes in a pot
T15	flag leaf	days	number of days from sowing to appearance of the flag leaf, observed in over 50% of plants

### Statistical analysis

Metabolomic observations were transformed using logarithmic transformation (log_2_y). For each trait separately, an analysis of variance was carried out in a model containing fixed effects of factors: genotype, time point, stress variant and the effects of their interaction. Significance of effects was declared at p ​​< 0.05 using the Bonferroni correction for multiple testing for 122 metabolites. The co-variability of phenotypic traits was tested using Pearson’s correlation coefficients, determining their statistical significance using the *t*-test. Statistical analyses as well as the corresponding graphs were performed in Genstat 19 (VSN Int., 2017). Integration of results was carried out using the correlation network method (Langfelder & Horvath, 2008, 2012) using the WGCNA package in the R system (β parameter = 1). Groups of strongly correlated features were determined by hierarchical grouping using the “complete link” method, with the parameters: cutHeight = 0.8, minSize = 5.

## Results

### Untargeted metabolomic analysis

Analysis allowed to detect 384 metabolites. Accumulation of 122 of them depended significantly on at least one of experimental factors and only these metabolites were taken for further analysis. Log_2_FC (Fold Change) values for all metabolites, genotypes and treatments, relative to C are given in S2 Table. Variance analysis revealed that metabolites concentration depended significantly on: genotype (76 compounds), time point (21) and environment (41), and on the interaction of genotype × time point (7), genotype × environment (29), and time point × environment (30) (S3 Table).

**Fig. 2:**
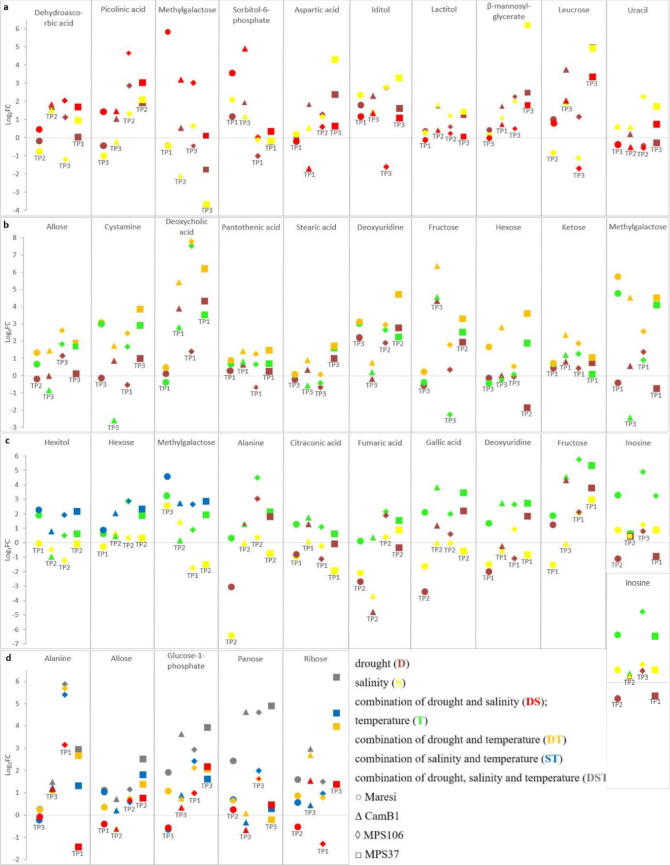
Effect of: **a** drought, salinity and their combination; **b** temperature, drought and their combination; **c** temperature, salinity and their combination; **d** combined stresses: DS, DT, ST, and DST on metabolite content Fold Change in different barley genotypes at selected TPs

We assessed mean impact of each environmental condition on metabolite accumulation (S2 Table), with a primary focus on the effect of combined stresses-relative to their individual stress components. The occurrence of DS generally led to higher accumulation of dehydroascorbic and picolinic acids, methylgalactose, and sorbitol-6-phosphate compared to when each stress was applied separately. However, the time point at which greater accumulation under DS was noted varied depending on the genotype (Fig. 2a). Conversely, the accumulation of aspartic acid (Asp), iditol, lactitol, β-mannosylglycerate (β-MG) was greater under separate D/S conditions (Fig. 2a). Considering effects with absolute value greater than 1 as considerably large, we note the effect of D for leucrose accumulation, whereas higher level of uracil under S was observed, and in both cases, the effect of DS was smaller (Fig. 2a). Meanwhile, DT had a stronger effect on the allose, cystamine, deoxycholic, pantothenic, and stearic acids, deoxyuridine, fructose, hexose, ketose, and methylgalactose accumulation compared to the individual stresses (Fig. 2b). ST mainly influenced the accumulation level of hexitol, hexose, and methylgalactose generally to a greater extent than when stresses were applied separated (Fig. 2c). However, among the single stresses, T generally led to the highest accumulation of alanine, citraconic, fumaric, and gallic acids, deoxyuridine, fructose, and inosine (Fig. 2c).

The most complex stress conditions - DST resulted in high accumulation level of alanine, allose, glucose-1-phosphate, panose, and ribose (Fig. 2d).

**Fig. 3:**
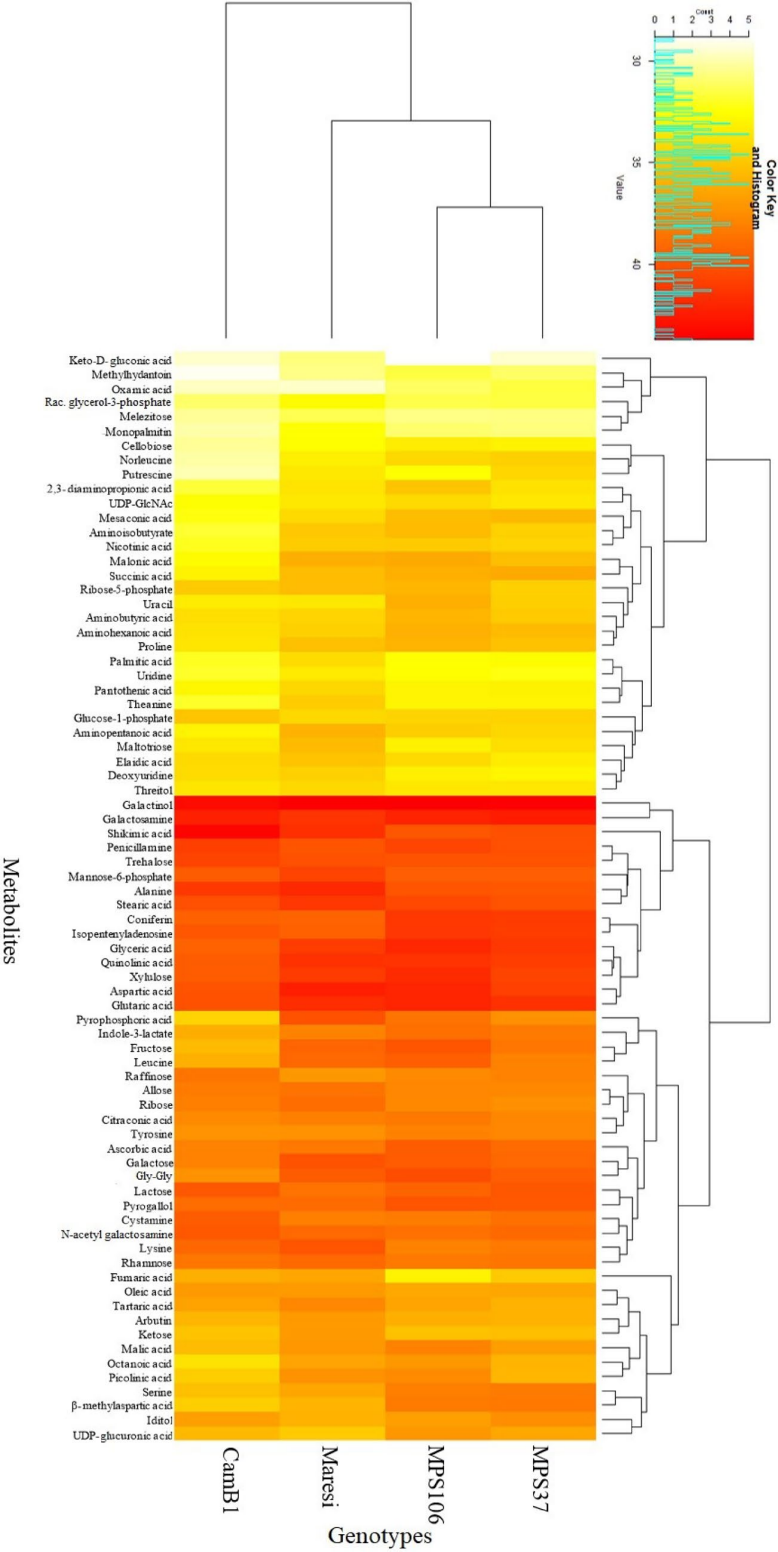
Mean accumulation of studied metabolites in different genotypes

Next, the average accumulation (across all treatments) of metabolites in each genotype was investigated (Fig. 3). The MPS genotypes were most distinctly differentiated from CamB1 by increased levels of putrescine, cellobiose, succinic, malonic, ascorbic, and mesaconic acids. Interestingly, MPS37 exhibited the highest levels of succinic and mesaconic acids, whereas MPS106 showed elevated levels of fructose and glycylglycine (Gly-Gly) compared to the other genotypes. Conversely, CamB1 primarily displayed increased levels of glucose-1-phosphate, shikimic acid, and raffinose, while Maresi had high levels of tartaric acid and lysine.

**Fig. 4:**
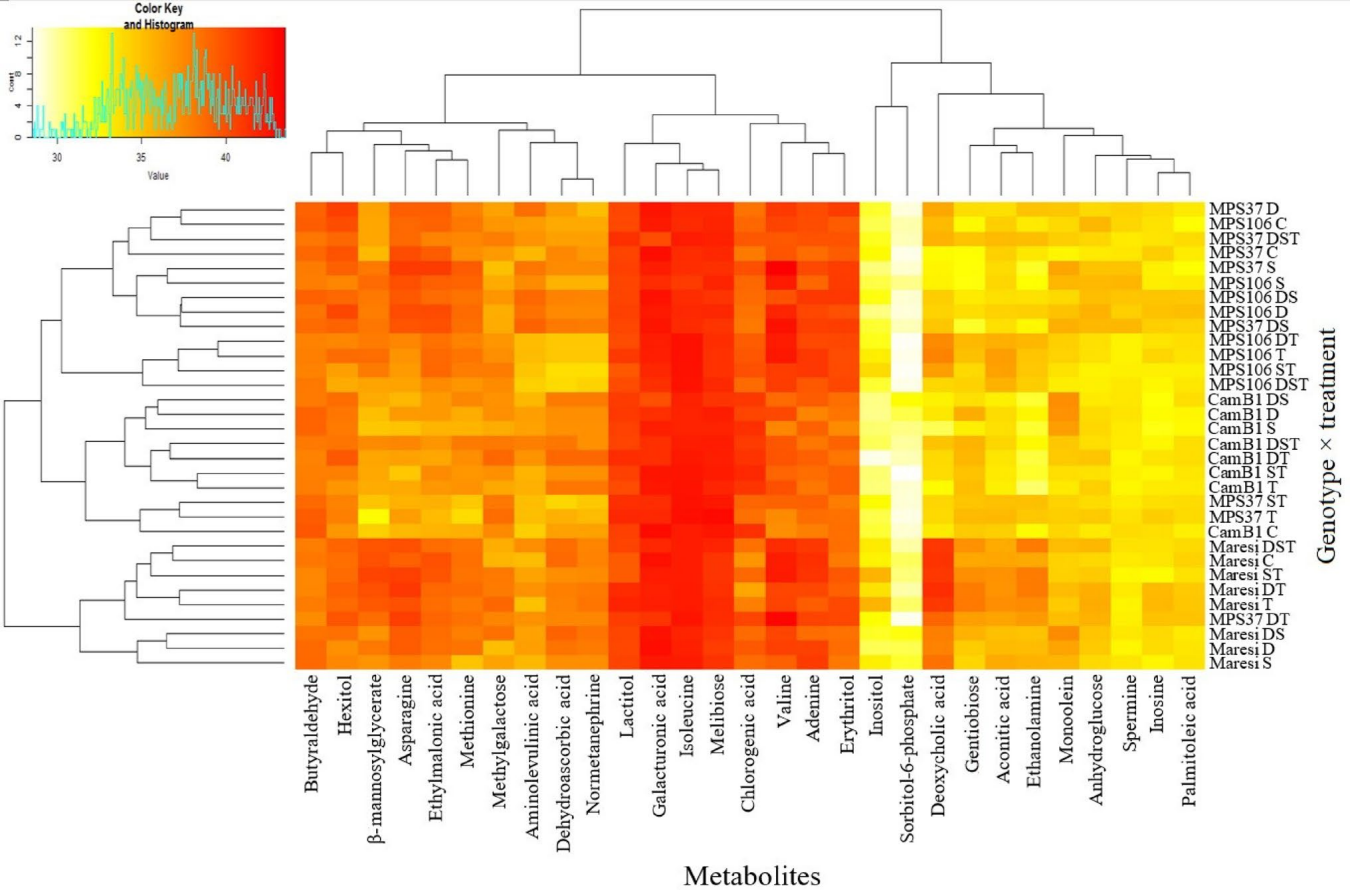
Mean accumulation of metabolites for genotype × abiotic stress combinations

Metabolites accumulation, which depended on genotype × environment interactions, is shown in Fig. 4. CamB1 consistently exhibited high content of chlorogenic acid and showed increases in monoolein and sorbitol-6-phosphate under D, S, and DS, with the highest increases recorded for the combined stress (S2 Table). In contrast, Maresi showed an increase of these metabolites mostly under DS, while maintaining a consistently high level of deoxycholic acid across all tested conditions. Additionally, T and its combinations generally caused an increase in gentiobiose and ethanolamine content across most of the analysed genotype × environment interactions.

An analysis of S2 Table showed that untargeted metabolomic analysis revealed three primary groups of compounds exhibiting the most pronounced stress-induced changes: amino acids, sugars, and organic acids. The fourth group encompassed other metabolites with substantial variations that could not be assigned to the aforementioned groups. Metabolites representing mentioned groups are discussed below.

The most intriguing change in proline (Pro) content was observed in CamB1 (especially at D, TP1), whereas in other genotypes, the most significant accumulation occurred at the later time point (TP3). In MPS106, D induced the highest Pro accumulation, while in MPS37, Pro increased mainly under S and DS stress. In Maresi, besides influence of DS, ST caused a nearly equal increase in Pro content. CamB1 showed elevated level of aminoisobutyrate at TP3 under D, S, DS. In CamB1 (TP1, TP2) and MPS106 (TP1) increase in glycylglycine was noted with the strongest effect of D. Asparagine level rose at TP1 in Maresi and CamB1, especially at D and DT. Among the MPS lines, variations in asparagine accumulation were found; specifically, MPS106 had higher content at TP3 compared to MPS37. Valine concentration was high in CamB1 at TP1 (beside DS) and TP2 (beside D or S), but decreased at TP3. Conversely, in MPS37 valine level were highest under S, DS, DT, and ST at TP3. At TP3, methionine sulfoxide level increased in CamB1 (under all stresses) and MPS lines (under D, S, DS, and DST). In Maresi, methionine sulfoxide content increased earlier - at TP1 at D, S, and DST conditions. The highest methionine content was found at TP1 in CamB1; generally, all genotypes showed increases under DS at TP1 and TP3 (along with D).

The greatest increase in carbohydrate content was observed in the MPS lines. While the most significant impact on raffinose accumulation in MPS37 was caused by ST at TP1 and TP2, in MPS106, an increase in raffinose was observed under all applied stresses at TP1. Maresi (TP2) and CamB1 (TP3) showed increased raffinose content under S, which contrasted with MPS37, where raffinose accumulation in response to single stresses (D or T, TP2) was noted and was markedly higher than under combined stress (DT). Conversely, under ST, raffinose accumulation exceeded that observed under the individual stresses (at TP2) in MPS37. An increase in fructose level was noted in MPS lines, mainly in response to T and its combinations (TP1) and, to a lesser extent at TP2 (except from ST). In CamB1 fructose level rose later (TP3) in mentioned conditions. Maresi and MPS lines generally were characterized by an increased hexose content mainly under T-related stresses (T, DT, ST, and DST) at TP1 and TP2. Notably, in MPS37 a major increase in hexose level was noted under all stress conditions at TP3. Additionally, MPS lines showed high levels of arbutin and rac. glycerol-3-phosphate during stress conditions involving T generally at all TPs.

Regarding organic acids, a trend was noted for palmitic acid in MPS106, with the highest increase noted at TP1 under stress involving T (ST, DT, DST) (S2 Table). At TP2, D, S and DS increased aminolevulinic acid content in the MPS lines, while all stresses elevated its level in CamB1. Additionally, S at TP2 increased level of quinic acid in MPS106. In turn, MPS37 showed the highest increases in elaidic, stearic, and tartaric acids mainly at TP3, with S exerting the strongest effect among single stress conditions, while DS had the least effect. The MPS lines at TP1 generally exhibited a high increase of gallic acid under D, T, DT, ST, DST.

Changes in the accumulation of other metabolites (the fourth category) were mostly noted in response to S, D, and DS, as across both MPS and CamB1 genotypes, with an increase in ascorbic acid (AsA) level at TP3. Maresi and MPS lines accumulated higher concentrations of polyamines with longer carbon chains, such as spermidine (SPD) and spermine (SPM). Elevated putrescine (PUT) level was observed at TP1 in MPS106, especially under D and DS, while MPS37 showed its highest accumulation under D or S.

### Phenotypic observations

**Fig. 5:**
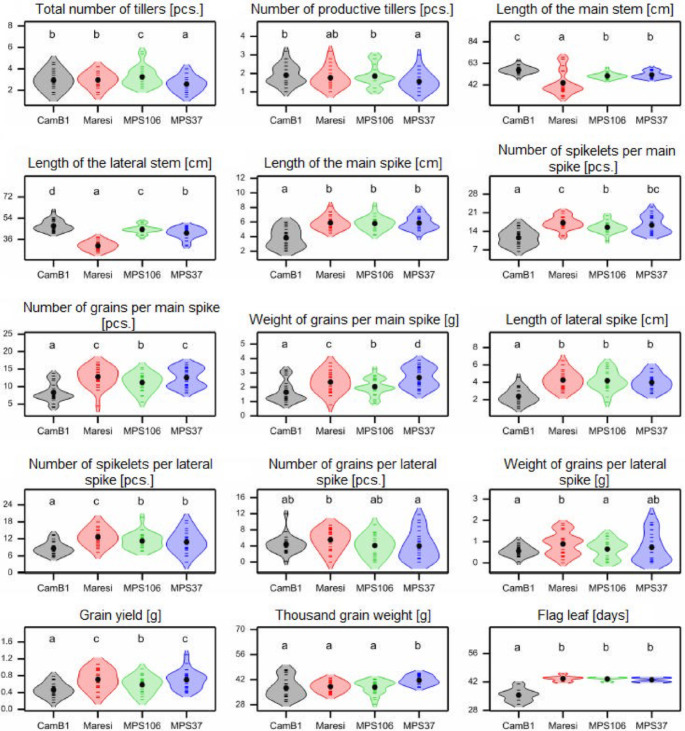
Distributions of the studied phenotypic traits in control and all analysed abiotic stress conditions. Mean values marked by dots, letters indicate statistically similar or different mean values according to the Fisher’s least significant difference test at the significance level of 0.05

MPS37 was characterized by the lowest total number of tillers (T1) (Fig. 5). The longest main stem (T3) and lateral stem (T4) were recorded for CamB1; however, due to environmental interactions, the longest main stem under control conditions was observed in Maresi (67.87 ± 0.57 cm). Both Maresi and MPS37 exhibited the highest number of spikelets per main spike (T6), number of grains in the main spike (T7), and weight of grain from the main spike (T8) (Fig. 5). Conversely, CamB1 displayed the lowest values of T6, T7, length of lateral spike (T9), grain yield (T13) and reached the flag leaf stage (39 BBCH) the earliest (T15).

**Fig. 6:**
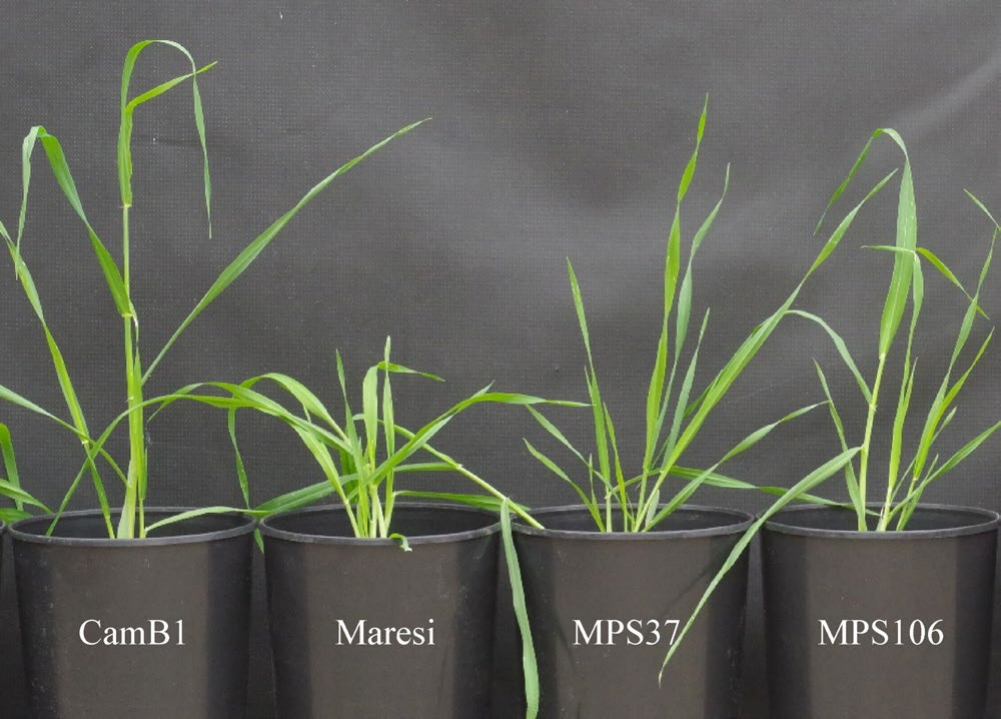
Phenotype of analysed barley genotypes in control conditions 30 days after sowing

**Fig. 7:**
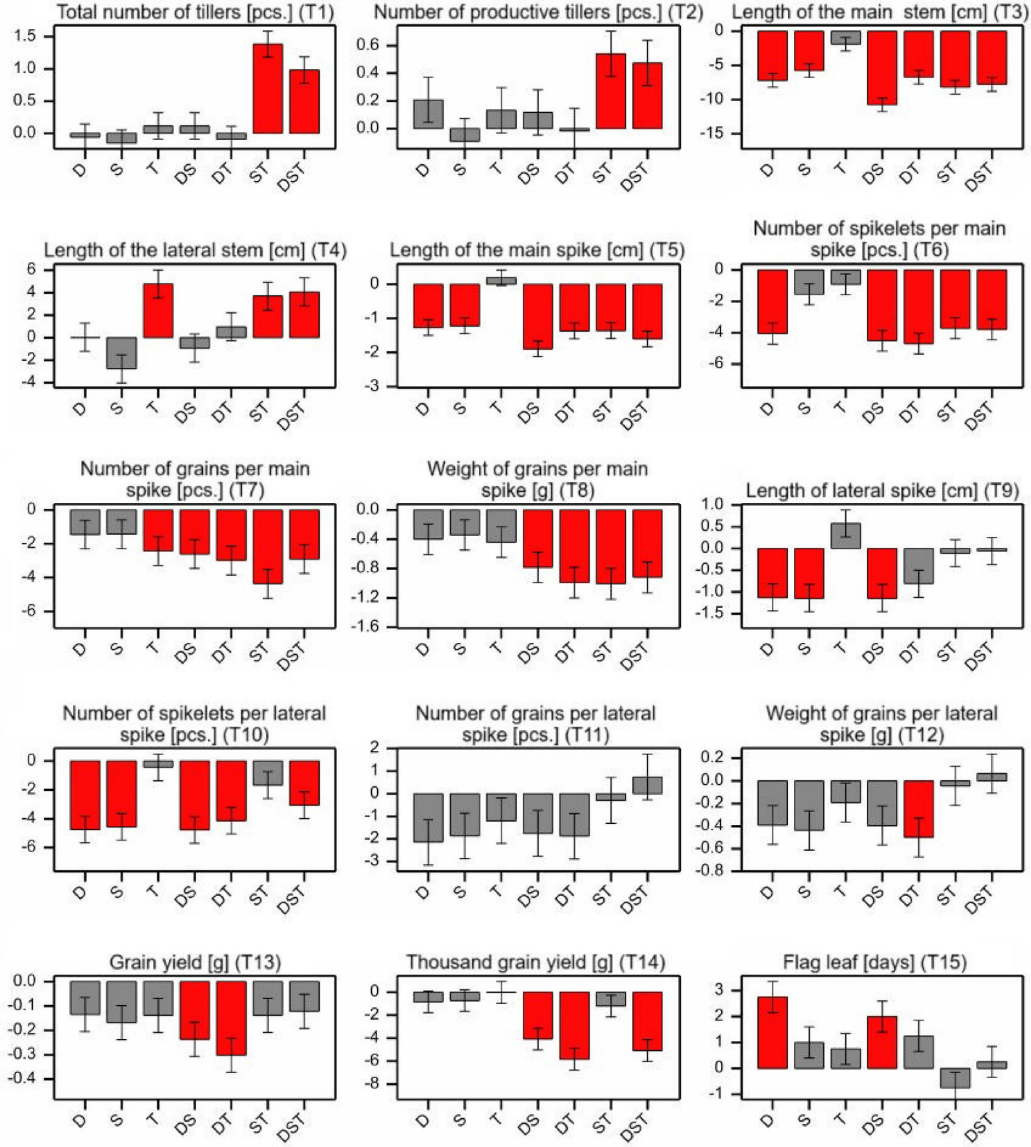
Contrasts assessment between mean values ​​of individual features for stress conditions versus control conditions; red bars indicate contrasts statistically significant at *p* < 0.05

The phenotypic response to abiotic stress conditions was primarily reflected by a decrease in trait values. Different effects of abiotic stresses relative to the control (Fig. 6) (averaged over genotypes) were observed across the analysed phenotypic features (Fig. 7). Except for T1, T2, T4, and T15, most stress effects were negative, indicating stress-induced reduction in phenotypic traits values. Contrast analysis revealed that the onset of the flag leaf stage (T15) was significantly delayed under D and DS. T stress had the least impact, decreasing only T7 and increasing T4. D, S, and DS generally exerted negative effects on most traits, although not always significantly - for example, T1, T2, T4, T11, and T12 showed non-significant decreases. Importantly, traits T1, T2, T8, T12, T13, and T14 were significantly negatively affected only under combined stress conditions. Additionally, the effects of D and S under DS conditions influenced traits T3, T5, and T7, whereas for T6, T9, and T10, the phenotypic response under DS more closely resembled responses to D alone. Notably, the values of T1, T2, T3, T6, T7, T8 were significantly higher under ST compared to T or S alone; however, in T4, T9, T10 the impact of T stress under ST was more pronounced. Under DT, the reduction in T7, T8, and T12-T14 were more substantial than under individual stresses. Conversely, the responses of traits T3, T5, T6, and T10 under DT resembled more closely those observed under D.

### Data integration

The correlation coefficients among the examined phenotypic traits are presented in Table 2. The strongest positive correlations were observed for T13, which showed significant relationships with most of other traits. Traits related to grain from the lateral spike (T11, T12) were highly positively correlated with each other, and a strong relationship between these traits and T13 was noted. Additionally, traits associated with the main spike (T7, T8) exhibited strong positive correlations with T13. The flag leaf stage showed negative correlation with T2, and with traits T11 through T13.

**Table 2:** Correlation coefficients of analysed phenotypic traits significant at *p* < 0.01

	T1	T2	T3	T4	T5	T6	T7	T8	T9	T10	T11	T12	T13	T14	T15
T1	1.00	*n*.s.	*n*.s.	*n*.s.	*n*.s.	*n*.s.	*n*.s.	*n*.s.	*n*.s.	*n*.s.	*n*.s.	*n*.s.	*n*.s.	*n*.s.	*n*.s.
T2	0.43	1.00	n.s.	n.s.	n.s.	n.s.	n.s.	n.s.	n.s.	n.s.	n.s.	n.s.	n.s.	n.s.	n.s.
T3	n.s.	n.s.	1.00	n.s.	n.s.	n.s.	n.s.	n.s.	n.s.	n.s.	n.s.	n.s.	n.s.	n.s.	n.s.
T4	0.25	n.s.	0.67	1.00	n.s.	n.s.	n.s.	n.s.	n.s.	n.s.	n.s.	n.s.	n.s.	n.s.	n.s.
T5	n.s.	n.s.	0.43	0.26	1.00	n.s.	n.s.	n.s.	n.s.	n.s.	n.s.	n.s.	n.s.	n.s.	n.s.
T6	n.s.	n.s.	0.39	n.s.	0.88	1.00	n.s.	n.s.	n.s.	n.s.	n.s.	n.s.	n.s.	n.s.	n.s.
T7	−0.25	0.28	0.33	n.s.	0.57	0.73	1.00	n.s.	n.s.	n.s.	n.s.	n.s.	n.s.	n.s.	n.s.
T8	n.s.	0.35	0.41	n.s.	0.51	0.64	0.89	1.00	n.s.	n.s.	n.s.	n.s.	n.s.	n.s.	n.s.
T9	n.s.	n.s.	0.35	0.31	0.81	0.74	0.48	0.41	1.00	n.s.	n.s.	n.s.	n.s.	n.s.	n.s.
T10	n.s.	n.s.	0.37	0.32	0.76	0.76	0.49	0.41	0.86	1.00	n.s.	n.s.	n.s.	n.s.	n.s.
T11	0.26	0.79	0.25	n.s.	n.s.	0.27	0.54	0.51	0.46	0.51	1.00	n.s.	n.s.	n.s.	n.s.
T12	0.29	0.79	0.24	n.s.	n.s.	0.25	0.50	0.56	0.46	0.51	0.95	1.00	n.s.	n.s.	n.s.
T13	n.s.	0.75	0.33	n.s.	0.38	0.45	0.74	0.80	0.47	0.50	0.86	0.89	1.00	n.s.	n.s.
T14	− 0.30	n.s.	n.s.	n.s.	n.s.	n.s.	0.24	0.57	n.s.	n.s.	n.s.	n.s.	0.32	1.00	n.s.
T15	n.s.	− 0.53	n.s.	n.s.	0.43	0.37	n.s.	n.s.	0.42	0.33	− 0.33	− 0.31	− 0.30	n.s.	1.00

n.s. - not statistically significant

Correlation analysis was done for phenotypic traits and metabolites on the basis of mean values for genotype × treatment combinations (Table 3). We identified four groups (G1-G4) composed of phenotypic traits and metabolites, that were most correlated. In G1 positive correlation of T4 with galactinol and negative with sorbitol-6-phosphate level was found. In G2 negative correlation of T1 and T2 with glyceric acid and phytol was noticed. In G3 the most negative correlation was found between T15 and shikimic acid level; meanwhile, T15 was positively correlated with PUT and 2,3-diphosphoglyceric (2,3-DPG) acid level. Within this group, it was also observed that the level of PUT and 2,3-DPG acid was positively correlated with yield characteristics (T7, T8, T13), which, in parallel, were negatively correlated with cystamine level. In turn, traits T5, T6, T9 and T10 (related to the length and number of spikelets of both main and lateral spikes) showed negative correlation with chlorogenic acid and lactitol content in group G4. Meanwhile, lateral spikes-related traits (T9, T10), had stronger positive correlation with aconitic and palmitic acids, inosine, monopalmitin and theanine content than for main spike (T5, T6).

**Table 3:** Correlation of metabolites and phenotypic traits analysed in the study (four groups G1–G4)

Group 1					
MIN − 0.5557; MAX 0.7168					
T3	1				
T4	0.51	1			
Galactinol	n.s.	0.67	1		
Monoolein	n.s.	− 0.46	n.s.	1	
Sorb-6-P	n.s.	− 0.56	− 0.55	0.72	1
	T3	T4	Galactinol	Monoolein	Sorb-6-P

Group 2						
MIN − 0.4723; MAX 0.6251						
T1	1					
T2	0.61	1				
Aminol. acid	n.s.	n.s.	1			
Anhydroglucose	n.s.	− 0.47	0.57	1		
Glyceric acid	− 0.42	− 0.44	0.63	0.52	1	
Phytol	− 0.42	− 0.47	0.46	0.59	0.51	1
	T1	T2	Aminol. acid	Anhydroglucose	Glyceric acid	Phytol

Group 3										
MIN − 0.7784; MAX 0.9223										
T7	1									
T8	0.92	1								
T13	0.8	0.81	1							
T15	0.5	n.s.	n.s.	1						
2,3-dipglyc. acid	0.54	0.5	0.55	0.52	1					
Adenine	0.42	n.s.	n.s.	0.56	n.s.	1				
Cystamine	− 0.6	−0.51	− 0.52	− 0.49	− 0.65	− 0.46	1			
N-acetyl galacto.	− 0.41	n.s.	n.s.	− 0.56	− 0.49	− 0.67	0.56	1		
Putrescine	0.56	0.51	0.53	0.65	0.72	n.s.	− 0.6	− 0.42	1	
Shikimic acid	− 0.5	− 0.43	n.s.	− 0.78	−0.58	− 0.52	0.54	0.67	− 0.64	1
	T7	T8	T13	T15	2,3-dipglyc. acid	Adenine	Cystamine	N-acetyl galacto.	Putrescine	Shikimic acid

Group 4													
MIN − 0.7528; MAX 0.9027													
T5	1												
T6	0.88	1											
T9	0.79	0.7	1										
T10	0.62	0.66	0.81	1									
Aconitic acid	0.43	0.42	0.68	0.63	1								
Aminopen. acid	0.52	0.56	0.51	0.54	0.68	1							
Chlorogenic acid	− 0.62	− 0.6	− 0.6	− 0.48	− 0.53	− 0.75	1						
Galactosamine	0.46	n.s.	0.41	n.s.	0.63	0.64	− 0.62	1					
Inosine	0.44	0.49	0.63	0.67	0.84	0.74	− 0.55	0.56	1				
Lactitol	− 0.49	− 0.46	− 0.52	− 0.55	− 0.55	-0.64	0.51	− 0.52	− 0.64	1			
Monopalmitin	n.s.	n.s.	0.59	0.65	0.9	0.73	− 0.55	0.5	0.79	− 0.68	1		
Palmitic acid	0.42	n.s.	0.55	0.59	0.87	0.63	− 0.56	0.6	0.84	− 0.57	0.84	1	
Theanine	0.46	0.41	0.6	0.54	0.9	0.62	− 0.5	0.56	0.79	− 0.57	0.88	0.84	1
	T5	T6	T9	T10	Aconitic acid	Aminopen. acid	Chlorogenic acid	Galactosamine	Inosine	Lactitol	Monopalmitin	Palmitic acid	Theanine

*Sorb-6-P* sorbitol-6-phosphate; *Aminol. acid* aminolevulinic acid; *2,3-DPG* 2,3-diphosphoglyceric acid; *N-ac. ga.* N-acetyl galactosamine; *PUT* putrescine; *Shi. a.* shikimic acid; *Acon. a.* aconitic acid; *Amin. a.* aminopentanoic acid; *Chlor. a.* chlorogenic acid; *Galacto.* galactosamine; *Monopal.* monopalmitin; *Palmitic a.* palmitic acid; *n.s.* not statistically significant

## Discussion

This study highlights the complex nature of barley’s response to simultaneous abiotic stresses, focusing on the unique, additive, synergy, and dominance effects described by Prasch and Sonnewald (2015). A key finding is the heavy reliance of these responses on genotype, underscoring the necessity for genotype-specific strategies in crop improvement. Our analyses revealed that most metabolites in barley accumulated uniquely under combined stress conditions, particularly influenced by genetic variations. For instance, the accumulation of sorbitol-6-phosphate and picolinic acid varied considerably among genotypes like Maresi, CamB1, and MPS lines, indicating diverse pathways for managing osmotic adjustment, ROS scavenging as well as carbon allocation (Dos Reis et al., 2012; Gao et al., 2018; Aucique-Pérez et al., 2019).

### Metabolic pathways and their functional roles

In the study we observed a differential role of metabolites, such as increase of dehydroascorbic acid (DHA) and methionine sulfoxide (MetO) under DS, and Asp along with β-MG showing highest increase in separately acting D and S stresses. DHA and MetO accumulation patterns underscore their role in ROS detoxification, with variations linked to genotype. We found the dominance of D in case of DHA accumulation in CamB1 and the synergy of D with S in MPS37. Since the AsA-DHA redox pair is one of the most fundamental ROS scavenging routes (Kaur et al., 2023), this DHA accumulation pattern in CamB1 may underlie its AsA utilization during D. Due to the synergistic rise in DHA, MPS37 may appear to be more suited to the DS combination; however, the high DHA content may also be maintained due to slower dehydroascorbate reductase action during DHA reduction (Jiang et al., 2022). Another important metabolomic pair of ROS scavengers is methionine (Met) and its sulfoxide (Liang et al., 2012; Savino et al., 2022). All analysed barley genotypes, under long-term D, S or DS increased MetO level, demonstrating the universality of this ROS degradation pathway in barley grown under osmotic stress. Moreover, CamB1 showed higher Met content at the beginning of stress action, indicating possibility of faster and more efficient ROS degradation compared to other analysed genotypes. The rapid amino acid accumulation in CamB1 suggests that its metabolome is highly plastic in response to abiotic stress, possibly as a result of protective mechanisms developed due to its origin. The β-MG content under stress reflects genotype-dependent gene expression variations, impacting osmotic balance and cellular macromolecule stabilization (Nobre et al., 2013). Higher levels of β-MG content were observed in response to S (MPS37) or D (CamB1). The presence of distinct allelic forms or differential expression of genes whose products impact rate of β-MG catabolism or anabolism, could be the reason of the increase in β-MG in a hyperosmotic environment brought on by a variety of stresses (D/S).

Elferjani et al. (2024) stated that elevated Asp content is significant during drought and recovery, potentially explaining increased Asp content in CamB1 leaves as its adaptation to growth under arid conditions, additionally allowing to faster recovery. The same authors proposed Asp as a biomarker of drought, on the other hand, Sadak et al. (2022) described the influence of Asp in alleviation of salinity in plants. In the present study we demonstrated elevated Asp level as a result of both stresses (D, S) suggesting its use as a dehydration/osmotic stress marker in barley; further, we propose citraconic and gallic acids as a T stress and leucrose as a D marker, respectively, as these stresses had the strongest effect on the mentioned metabolites across all tested barley genotypes.

### Temperature stress as a differentiating factor

Temperature stress, among drought or salinity, emerged as a critical factor influencing various components like fumaric and tartaric acids, with significant differences noted across genotypes in terms of metabolomic adjustment. Fumaric acid acts in OA, helping to maintain cell turgor and water retention under osmotic stresses (Iglesias-Moya et al., 2023). However, under stress where photosynthesis is inhibited (such as T), plants may use organic acids like fumarate as a carbon and energy reserve, because it acts as a metabolic bridge between glycolysis, TCA cycle, and anaplerotic reactions. Its high content in T stress might be a result of increased metabolic rates as respiration, oxidative stress or TCA cycle alternations – enzymatic activities in the TCA cycle are temperature sensitive (such as fumarase activity) (Zhou et al., 2025), potentially affecting fumaric acid level in leaves of stressed barley plants. We observed generally unique DT effect in case of tartaric acid an exception being MPS37, where a synergistic interaction of the stresses on this metabolite was most likely present. Tartaric acid is considered a phenolic antioxidant that protects the photosynthetic apparatus and induces catalase and glutathione peroxidase activity (Hýsková et al., 2017; Singh et al., 2017; Yildiztugay et al., 2017). It may also mediate in the shikimic acid metabolic pathway and contribute to the biosynthesis of protective compounds (Quan et al., 2016; Bagues et al., 2019; Zheng et al., 2019). The collected observations indicate that metabolic adaptation to thermal stress, exhibits strong genotypic variation and is different than adaptations observed in osmotic stress (D, S) action. Key organic compounds, such as fumaric and tartaric acids, play multifunctional protective and energetic roles (Mibei et al., 2018; Hassanein et al., 2021), thereby shaping the physiological plasticity of barley plants.

Under combined stress containing T, certain genotypes, like MPS lines, exhibited enhanced carbohydrate accumulation, particularly of hexoses, which appears to act as a universal mechanism for temperature fluctuation mitigation. Applied ST stress mostly led to the unique accumulation of hexoses in all genotypes. However, generally in all combined stress conditions involving T stress, hexoses content, including fructose, increased, which may represent a universal barley mechanism to mitigate such stress conditions. It is worth noting that T had the strongest effect on fructose accumulation among all stress treatments. The protective role of hexoses on plants in abiotic stress is well known (Sami et al., 2016; Jiménez-Arias et al., 2021), thus genotypes presenting their higher content are better adapted to sustain temperature fluctuations. The genotype-specific response in term of ketose accumulation suggests an osmoprotective role, with variations pointing to differences in synthetic pathways and substrate availability. Interestingly, ketose accumulation under DT was synergistic in case of CamB1 line, and unique in the other genotypes. Ketoses have mainly osmoprotective functions (Serrano et al., 2019), thus their synergistic accumulation pattern in Syrian genotype may be based on its origin, as an adaptive mechanism. However, it should be kept in mind that the occurrence of various ketoses in leaves may result from different availability of substrates for their synthesis or existing differences in their isomerization in different genotypes (Gemmer et al., 2021). MPS genotypes also responded to single or combined T stress by increased arbutin content. Such results support Lawas et al. (2019) and Janda et al. (2021) results, because arbutin performs protective function towards membranes, inhibiting phospholipases in dehydrated cells. It is worth adding that T had the greatest impact on increased levels of free fatty acids in the tested barley genotypes, as observed in our previous lipidomic study (Kuczyńska et al., 2019), probably due to changes within cell membranes composition.

Early carbohydrates accumulation in MPS lines may result in rapid activation of protective mechanisms in response to stress and faster carbon compounds mobilization regulating turgor, ROS level and stabilizing cellular macromolecules. CamB1 originating from arid region, may have mechanisms that slow down dehydration and only after prolonged exposure to stress conditions it triggers accumulation of carbohydrate compounds.

### Phenotypic implications and stress management in barley

Early plant responses to stress are highly dynamic and may undergo substantial reprogramming during subsequent developmental phases. As a consequence, multiple intervening processes, including stress recovery capacity, compensatory growth, developmental plasticity, and genotype-specific resilience, may modulate how early metabolic changes are ultimately reflected in mature-stage phenotypic traits. Nevertheless, beyond characterizing metabolic alterations across multiple stress conditions and barley genotypes, our objective was to identify early metabolic signatures associated with long-term stress consequences. Such associations may serve as predictive indicators of stress impact and genotype-specific sensitivity.

Abiotic stresses, especially most often occurring in nature - DT combination (Zandalinas et al., 2016, 2017), significantly reduced phenotypic traits, confirming the synergistic and, in some cases dominant effect (e.g. T3, T5, T6) of combined stresses over individual ones. A greater decrease in such traits under DT is consistent with Rollins et al. (2013), suggesting a larger (additive or synergistic) effect of combined stresses on reduction of phenotypic traits. Such differences may be based on the water availability during T stress, because as Rizhsky et al. (2004) and Posch et al. (2019) shown, under DT, stomata close to protect against excessive transpiration, limiting CO_2_ assimilation. What is more, our previous transcriptomic study on barley flag leaf exposed to D, T, and DT demonstrated that, D is the dominant factor affecting gene expression during DT stress (Mikołajczak et al., 2023). In case of ST, T reduced phenotypic traits to lesser extent than S, resulting in significant increase in some of them (T1, T2, T6, T8) compared to single stress influence, making such reaction unique. In some cases, dominance of T stress over S was noted (T4, T7), however, in the other, S was dominant stress over T under ST (T3, T5), highlighting the need to perform analyses of how combined abiotic stress influence barley’s phenotypic and yield-forming features in comparison to solitary ones.

Our assessment of phenotypic traits underlines the dominant and synergistic responses elicited by these stressors, with metabolites like galactinol and PUT playing crucial roles in improving yield traits and flag leaf development under constrained conditions. These metabolic adjustments likely enhance photosynthetic efficiency and CO_2_ assimilation, as suggested by the correlation with 2,3-DPG level. Moreover, galactinol is a substrate in raffinose family oligosaccharides (RFO) synthesis, which are crucial regulators of plant development and stress response (Yan et al., 2022). Function of 2,3-DPG in yield-shaping is probably based on its indirect participation in sugar metabolism, starch synthesis, protection of photosynthetic apparatus, and carbon storage, explaining correlation observed in our study (Rychter & Rao, 2005). In relation to PUT, numerous studies have confirmed that foliar application of polyamines increases the yield of various plants under abiotic stress. This effect is associated with an increased content of photosynthetic pigments, which in turn enhances the rate of photosynthesis. Additionally, polyamines had positive effect on OA, water and Na^+^/K^+^ ratio, and ROS detoxification (Saleethong et al., 2013; Torabian et al., 2018; Abd Elbar et al., 2019).

## Conclusions

This comprehensive exploration of barley’s response to abiotic stresses affirms the importance of genotype-specific approaches for effective stress mitigation. Our metabolites profiling revealed different metabolic response strategies to combined stresses depending on the genotype and metabolite type, but the unique metabolic signatures identified in this study, such as the role of specific amino acids and sugars, provide valuable insights into physiological resilience mechanisms. The findings propose potential biomarkers for stress breeding, including aspartic acid for osmotic stress and leucrose for drought. By managing these metabolic pathways, breeders could enhance barley’s stress tolerance and productivity, ultimately contributing to more resilient agricultural practices. These insights into the metabolic bottlenecks and enhancement strategies illuminate paths for improving barley performance under increasingly variable climate conditions.

## Supplementary Information

Below is the link to the electronic supplementary material.

Supplementary Material 1—S1 Table Normalized GC-MS data.

Supplementary Material 2—S2 Table Log_2_FC of metabolite level in relation to control conditions.

Supplementary Material 3—S3 Table Metabolites which accumulation depended on genotype, time point, environment or the interaction of these factors (ANOVA, *F* test at *p* < 0.05).

## Data Availability

Data is provided within the manuscript or supplementary information files.

## Abbreviations

2,3-DPG: 2,3-diphosphoglyceric acid
AsA: Aspartic acid
Asp: Aspartic acid
BBCH: Two-digit code scale precisely defining all phenological growth stages of plants
C: Control conditions
D: Drought
DHA: Dehydroascorbic acid
DS: Combination of drought and salinity
DST: Combination of drought, salinity and temperature stress
DT: Combination of drought and temperature stress
FC: Fold change
FWC: Field water capacity
G1-G4: Correlation group (1-4) of metabolites and phenotypic traits
Gly-Gly: Glycylglycine
Met: Methionine
MetO: Methionine sulfoxide
OA: Osmotic adjustment
Pro: Proline
PUT: Putrescine
RFO: Raffinose family oligosaccharides
RN/OS: Reactive nitrogen/oxygen species
ROS: Reactive oxygen species
Rt: Retention time
S: Salinity
SPD: Spermidine
SPM: Spermine
ST: Combination of salinity and temperature stress
T: Temperature stress
T1-T15: Abbreviated phenotypic traits (detailed in table 1)
TCA: Tricarboxylic acid cycle
TIC: Total ion current
TP1-TP3: Time point 1-3
β-MG: β-mannosylglycerate
