# Supplementary material for: Deciphering barley’s stress response: metabolomic strategies and phenotypic implications under multiple abiotic stresses
Source: Metabolomics. 2026 Mar 7;22(2):36. doi: 10.1007/s11306-026-02406-8 (PMC12967591; doi:10.1007/s11306-026-02406-8)
Supplement: Supplementary file 3 — Supplementary Material 3—S3 Table Metabolites which accumulation depended on genotype, time point, environment or the interaction of these factors (ANOVA, F test at p < 0.05). [file 11306_2026_2406_MOESM3_ESM.docx]

S3 Table Metabolites which accumulation depended on genotype, time point, environment or the interaction of these factors (ANOVA, *F­* test at p < 0.05)

| Compound | Genotype (G) | Time point (TP) | Environment (E) | G × TP | G × E | TP × E |
| --- | --- | --- | --- | --- | --- | --- |
| 2,3-diphosphoglyceric acid | - | - | - | ✓ | - | ✓ |
| 2,3-diaminopropionic acid | ✓ | - | - | - | - | ✓ |
| Aconitic acid | - | - | - | ✓ | ✓ | - |
| Adenine | - | - | - | - | ✓ | - |
| Alanine | ✓ | ✓ | ✓ | - | - | - |
| Allose | ✓ | - | ✓ | - | - | - |
| Aminobutyric acid | ✓ | - | - | - | - | ✓ |
| Aminohexanoic acid | ✓ | - | - | - | - | ✓ |
| Aminoisobutyrate | ✓ | - | - | - | - | - |
| Aminolevulinic acid | - | - | - | - | ✓ | - |
| Aminopentanoic acid | ✓ | - | - | - | - | ✓ |
| Anhydroglucose | - | - | - | - | ✓ | - |
| Arbutin | ✓ | ✓ | ✓ | - | - | - |
| Ascorbic acid | ✓ | - | - | - | - | ✓ |
| Asparagine | - | ✓ | - | - | ✓ | - |
| Aspartic acid | ✓ | - | - | - | - | ✓ |
| Butyraldehyde | - | - | - | - | ✓ | - |
| Cellobiose | ✓ | - | - | - | - | - |
| Chlorogenic acid | - | - | - | - | ✓ | - |
| Citraconic acid | ✓ | - | - | - | - | - |
| Coniferin | ✓ | - | - | - | - | - |
| Cystamine | ✓ | - | ✓ | - | - | - |
| Dehydroascorbic acid | - | - | - | - | ✓ | - |
| Deoxycholic acid | - | - | - | ✓ | ✓ | ✓ |
| Deoxyuridine | ✓ | ✓ | ✓ | - | - | - |
| Elaidic acid | ✓ | ✓ | ✓ | - | - | - |
| Erythritol | - | - | - | - | ✓ | - |
| Ethanolamine | - | - | - | - | ✓ | ✓ |
| Ethylmalonic acid | - | - | - | - | ✓ | ✓ |
| Fructose | ✓ | - | - | - | - | ✓ |
| Fumaric acid | ✓ | - | ✓ | - | - | - |
| GABA | - | - | - | - | - | ✓ |
| Galactinol | ✓ | - | ✓ | - | - | - |
| Galactosamine | ✓ | - | ✓ | - | - | - |
| Galactose | ✓ | - | - | - | - | - |
| Galacturonic acid | - | - | - | - | ✓ | - |
| Gallic acid | - | - | ✓ | - | - | - |
| Gentiobiose | - | ✓ | - | - | ✓ | - |
| Glucose-1-phosphate | ✓ | - | ✓ | - | - | - |
| Glucuronic acid | - | - | ✓ | - | - | - |
| Glutaric acid | ✓ | - | - | - | - | ✓ |
| Glyceric acid | ✓ | - | - | - | - | ✓ |
| Gly-Gly | ✓ | - | - | - | - | ✓ |
| Hexitol | - | ✓ | - | - | ✓ | - |
| Hexose | - | - | ✓ | - | - | - |
| Homocysteine | - | - | ✓ | ✓ | - | - |
| Iditol | ✓ | - | - | - | - | - |
| Indole-3-lactate | ✓ | - | - | - | - | - |
| Inosine | - | - | - | - | ✓ | ✓ |
| Inositol | - | ✓ | - | - | ✓ | - |
| Isoleucine | - | - | - | - | ✓ | - |
| Isopentenyladenosine | ✓ | - | - | - | - | - |
| Keto-D-gluconic acid | ✓ | ✓ | - | - | - | - |
| Ketose | ✓ | ✓ | ✓ | - | - | - |
| Lactitol | - | - | - | - | ✓ | - |
| Lactose | ✓ | - | - | - | - | - |
| Leucine | ✓ | ✓ | - | - | - | - |
| Leucrose | - | - | ✓ | - | - | - |
| Lysine | ✓ | - | ✓ | - | - | - |
| Malic acid | ✓ | - | - | - | - | ✓ |
| Malonic acid | ✓ | - | - | - | - | - |
| Maltose | - | - | ✓ | - | - | - |
| Maltotriose | ✓ | - | - | - | - | - |
| Mannose-6-phosphate | ✓ | ✓ | ✓ | - | - | - |
| Melezitose | ✓ | - | - | - | - | ✓ |
| Melibiose | - | - | - | - | ✓ | - |
| Mesaconic acid | ✓ | - | - | - | - | ✓ |
| Methionine | - | ✓ | - | - | ✓ | - |
| Methionine sulfoxide | - | - | ✓ | - | - | - |
| Methylgalactose | - | - | - | - | ✓ | ✓ |
| Methylhydantoin | ✓ | - | - | - | - | ✓ |
| Monoolein | - | - | - | - | ✓ | - |
| Monopalmitin | ✓ | - | ✓ | - | - | - |
| N-acetyl galactosamine | ✓ | - | ✓ | - | - | - |
| Nicotinic acid | ✓ | - | - | - | - | - |
| Norleucine | ✓ | - | - | - | - | - |
| Normetanephrine | - | - | - | - | ✓ | - |
| Octanoic acid | ✓ | - | - | - | - | ✓ |
| Oleic acid | ✓ | - | ✓ | - | - | - |
| Ornithine | - | ✓ | - | - | - | - |
| Oxamic acid | ✓ | - | - | - | - | ✓ |
| Palmitic acid | ✓ | - | ✓ | - | - | - |
| Palmitoleic acid | - | - | - | - | ✓ | - |
| Panose | - | - | ✓ | - | - | - |
| Pantothenic acid | ✓ | - | ✓ | - | - | - |
| Penicillamine | ✓ | - | ✓ | - | - | - |
| Phytol | - | - | ✓ | - | - | - |
| Picolinic acid | ✓ | - | - | - | - | - |
| Proline | ✓ | - | - | - | - | - |
| Putrescine | ✓ | - | - | - | - | - |
| Pyrogallol | ✓ | - | ✓ | - | - | - |
| Pyrophosphoric acid | ✓ | - | - | - | - | - |
| Quinic acid | - | ✓ | - | - | - | - |
| Quinolinic acid | ✓ | - | - | - | - | ✓ |
| Rac. glycerol-3-phosphate | ✓ | - | ✓ | - | - | - |
| Raffinose | ✓ | - | ✓ | - | - | - |
| Rhamnose | ✓ | ✓ | ✓ | - | - | - |
| Ribitol | - | - | ✓ | - | - | - |
| Ribose | ✓ | - | ✓ | - | - | - |
| Ribose-5-phosphate | ✓ | ✓ | - | - | - | - |
| Serine | ✓ | - | - | - | - | ✓ |
| Shikimic acid | ✓ | - | ✓ | - | - | - |
| Sorbitol-6-phosphate | - | - | - | - | ✓ | - |
| Spermidine | - | - | ✓ | - | - | - |
| Spermine | - | - | - | ✓ | ✓ | ✓ |
| Stearic acid | ✓ | - | - | - | - | - |
| Succinic acid | ✓ | - | - | - | - | - |
| Tartaric acid | ✓ | ✓ | ✓ | - | - | - |
| Theanine | ✓ | - | ✓ | - | - | - |
| Threitol | ✓ | - | - | - | - | ✓ |
| Trehalose | ✓ | - | ✓ | - | - | - |
| Tyramine | - | - | - | ✓ | - | - |
| Tyrosine | ✓ | - | - | - | - | - |
| UDP-GlcNAc | ✓ | - | - | - | - | ✓ |
| UDP-glucuronic acid | ✓ | - | ✓ | - | - | - |
| Uracil | ✓ | - | - | - | - | ✓ |
| Uridine | ✓ | ✓ | - | - | - | - |
| Valine | - | - | - | ✓ | ✓ | ✓ |
| Xylono-1,5-lactone | - | ✓ | ✓ | - | - | - |
| Xylulose | ✓ | - | - | - | - | - |
| β-mannosylglycerate | - | ✓ | - | - | ✓ | - |
| β-methylaspartic acid | ✓ | - | - | - | - | - |
